# Experimental Investigation of the Viscosity and Stability of Scleroglucan-Based Nanofluids for Enhanced Oil Recovery

**DOI:** 10.3390/nano14020156

**Published:** 2024-01-10

**Authors:** Rubén H. Castro, Laura M. Corredor, Sebastián Llanos, María A. Causil, Adriana Arias, Eduar Pérez, Henderson I. Quintero, Arnold R. Romero Bohórquez, Camilo A. Franco, Farid B. Cortés

**Affiliations:** 1 Grupo de Investigación en Fenómenos de Superficie—Michael Polanyi, Facultad de Minas, Universidad Nacional de Colombia—Sede Medellín, Medellín 050034, Colombia; macausill@unal.edu.co (M.A.C.); caafrancoar@unal.edu.co (C.A.F.); fbcortes@unal.edu.co (F.B.C.); 2 Centro de Innovación y Tecnología—ICP, Ecopetrol S.A., Piedecuesta 681011, Colombia; laura.corredor@ecopetrol.com.co (L.M.C.); henderson.quintero@ecopetrol.com.co (H.I.Q.); 3 Grupo de Investigación en Química Estructural (GIQUE), Escuela de Química, Universidad Industrial de Santander, Bucaramanga 680006, Colombia; sllanosg@unal.edu.co (S.L.); adriana.ariasn@gmail.com (A.A.); arafrom@uis.edu.co (A.R.R.B.); 4 Departamento de Ingeniería Mecánica, Universidad Francisco de Paula Santander, Ocaña 546551, Colombia; eeperezr@ufpso.edu.co

**Keywords:** biopolymer, scleroglucan, nanofluids, viscosity behavior, enhanced oil recovery

## Abstract

Biopolymers emerge as promising candidates for enhanced oil recovery (EOR) applications due to their molecular structures, which exhibit better stability than polyacrylamides under harsh conditions. Nonetheless, biopolymers are susceptible to oxidation and biological degradation. Biopolymers reinforced with nanoparticles could be a potential solution to the issue. The nanofluids’ stability and performance depend on the nanoparticles’ properties and the preparation method. The primary objective of this study was to evaluate the effect of the preparation method and the nanoparticle type (SiO_2_, Al_2_O_3_, and TiO_2_) on the viscosity and stability of the scleroglucan (SG). The thickening effect of the SG solution was improved by adding all NPs due to the formation of three-dimensional structures between the NPs and the SG chains. The stability test showed that the SG + Al_2_O_3_ and SG + TiO_2_ nanofluids are highly unstable, but the SG + SiO_2_ nanofluids are highly stable (regardless of the preparation method). According to the ANOVA results, the preparation method and standing time influence the nanofluid viscosity with a statistical significance of 95%. On the contrary, the heating temperature and NP type are insignificant. Finally, the nanofluid with the best performance was 1000 ppm of SG + 100 ppm of SiO_2__120 NPs prepared by method II.

## 1. Introduction

Enhanced oil recovery (EOR) is becoming essential in the global oil supply because production from mature fields is declining and hydrocarbon discoveries are insufficient to meet the growing energy demand [1,2,3]. EOR contributes to maximizing oil reserves, extending the life of fields, and increasing the oil recovery factor. Flooding with water-soluble synthetic polymers, mainly with partially hydrolyzed polyacrylamide (HPAM), has been used to improve oil sweep efficiency by reducing water mobility and water permeability in the swept zone and by diverting the displacing fluid to unswept zones [1,4,5,6,7]. However, synthetic polymers are affected by reservoir temperature, formation water salinity, and hardness [8]. Polysaccharides such as xanthan gum (XG) [9], carboxymethylcellulose (CMC) [10], schizophyllan (SPG) [11], and scleroglucan (SG) [12] have emerged as an alternative to HPAM due to their remarkable rheological properties and resistance to hydrolysis, pH, electrolytes, mechanical shearing, and temperature [13,14].

Scleroglucan is a non-ionic, water-soluble polysaccharide produced by Sclerotium species [12,15,16,17,18]. It can be made with different branching frequencies, side-chain lengths, and molecular weights (ranging from 1.3–3.2·105 to 0.3–6.0·106 Da) depending on the fermentation conditions, the producing species (Sclerotium glucanicum, Sclerotium rolfsii, and Sclerotium delphinii), and the extraction methods [19,20,21]. Sclerotium glucanicum and Sclerotium rolfsii are the main species used for SG production. SG forms a triple-helical structure when dissolved in water. These solutions exhibit a shear thinning behavior but can tolerate high temperatures, a pH range of 1 to 11, and various electrolytes. The rate at which the viscosity of the SG solutions develops depends on purity grade, mixing, temperature, pH, and polymer concentration. Previous studies have shown that improper preparation of the SG solutions contributes to the negative performance of this biopolymer at laboratory and field scales (low viscosity, poor filterability, and formation damage) [15,16,22].

In the last decade, the combination of polymer flooding and nanoparticles (NPs) has been investigated as a promising method to enhance oil recovery through the improvement of the rheological properties of the injected fluid [23,24], reductions in polymer retention and oil-water interfacial tension [25,26], and wettability alteration [23,27,28,29,30,31,32]. The NPs that have shown great potential to enhance oil recovery for both light and heavy oil are SiO_2_, TiO_2,_ and Al_2_O_3_ NPs [33,34]. The NPs can be dispersed in the polymer solution [34,35,36], or the polymer chains can be grafted onto the NP surface [33,37,38,39].

The nanofluids can be prepared by mixing following one-step or two-step methods. The one-step method consists of simultaneously synthesizing and dispersing the nanoparticles into a fluid. In the two-step method, the nanoparticles are first synthesized and then dispersed into the fluid [40]. Employing the mixing of biopolymers with metal oxide NPs for heavy oil recovery, Corredor et al. [23] reported that adding untreated silica, SiO_2_-MPS, and SiO_2_-OTES NPs improved the thickening behavior of XG solutions. In contrast, Fe(OH)_3_, Al_2_O_3_, and TiO_2_ NPs decreased the viscosity of the biopolymer solutions. At 0 ppm and 3000 ppm NaCl, the NPs increased the cumulative oil recovery between 3% and 9% and between 1% and 5%, respectively. However, at 10,000 ppm NaCl, only Fe(OH)_3_ and TiO_2_ NPs increased the cumulative oil recovery between 2% and 3%. The differences in the performance of the nanofluids were ascribed to the changes in the electrostatic interactions between NPs-XG-counterions-sand grains. Similarly, Saha and coworkers [41] found that the incorporation of hydrophilic SiO_2_ NPs into the XG solutions reduced the oil-water IFT, increased the viscosity of the biopolymer solution, stabilized the emulsions, and changed the wettability of the porous media, leading to an increment in the cumulative oil recovery of 20.82% at 30 °C and 18.44% at 80 °C as compared withwater flooding.

Rellegadla et al. [36] reported that nickel-assisted XG flooding yielded the highest recovery of 5.98% residual oil in place (ROIP), compared with 4.48% ROIP of XG flooding and 4.58% ROIP of NP flooding due to the higher intrinsic viscosity of the nickel-XG nanofluid. Orodu et al. [30,35] studied the effect of Al_2_O_3_ NPs (30–60 nm) on the performance of the Potato Starch (PSP) and the Gum Arabic (GA). After waterflooding, they reported an incremental oil recovery between 5–12% and 5–7% for the PSP-NPs and the GA-NPs samples. These results were attributed to the increment in the biopolymer’s viscosity and the improvement of their thermal stability caused by the alumina NPs.

In a later work, Rueda et al. [31] evaluated the effect of modified silica nanoparticles (1000 ppm of polymer-coated silica nanoparticles) on the performance of XG and SG solutions (160 and 250 ppm). The results showed that NP-assisted Xanthan flooding achieved the highest ultimate oil recovery at all evaluated conditions due to a more homogenous dispersion of the NPs in the XG solution and reduced polymer adsorption. The dispersion of the NPs in the SG solution was unsuccessful. Buitrago et al. [42] evaluated the effect of the sonication time, the addition order of the components, and the polymer hydration time on the rheological behavior of XG-hydrophilic SiO_2_ nanofluids. They concluded that the preparation method has little impact on the performance of the nanofluids because all of them exhibited similar rheological behavior and viscosity values.

The two-step method is used in industries to produce nanofluids on a large scale due to its lower production cost than the one-step method. However, this method is challenging to avoid the agglomeration of NPs. No literature investigations have described a specific method to prepare a scleroglucan-based nanofluid and nanoparticles by an easy and practical method that takes advantage of the mechanical effect to solubilize the biopolymer and disperse the nanoparticles [43]. Previous reports, such as Rueda [31] and Buitrago [42], have used lower-purity biopolymer solutions (SG and XG) to prepare nanofluids using magnetic effect and ultrasound (300 W) with long hydration times (24 h, full hydration for 7 days or longer) with no representative viscosity differences, in contrast to this study using a high-performance immersion blender at 20,000 rpm [44,45] to represent a practical setting on a possible field scale and to avoid low hydration effects in EOR polymer preparation [22,31,46]. For this reason, the primary objective of this study is to examine the feasibility of improving the stability and viscosity of SG-based nanofluids prepared by an easy two-step method for EOR applications by changing the preparation method and the NP type.

## 2. Materials and Methods

### 2.1. Materials and Reagents

The biopolymer employed was a commercial EOR-grade scleroglucan (SG, purity >99%, 5% humidity) with a molecular weight of 4 × 10^6^ Da. For the preparation of the synthetic brine, 0.83 g/L sodium chloride (NaCl, 99.5% pure, Merck Millipore, Burlington, MA, USA), 0.04 g/L potassium chloride (KCl, 99.5% pure, Merck Millipore, USA), 0.07 g/L magnesium chloride (MgCl_2_.6H_2_O, 99% pure, Merck Millipore, USA), and 0.34 g/L calcium chloride (CaCl_2_.2H_2_O, 99% pure, Merck Millipore, USA), and type II water (pH ≈ 7) were used. Commercial nanoparticles of SiO_2_, Al_2_O_3_, and TiO_2_ of different nature, sizes, and surface areas was used for the nanofluid preparations as described in Table 1.

### 2.2. Methods

#### 2.2.1. Nanofluid Preparation

The brine was prepared in deionized water and filtered through a 0.45 µm MCE membrane filter (Merck Millipore, USA) before use. The biopolymer solution was made as proposed by Abraham and Sumner [43] and Castro et al. [44,45]. The nanofluids were prepared at a fixed concentration of 1000 ppm SG and 100 ppm NPs, following four different methodologies (I, II, III, IV) to determine the effect of the nanofluid preparation on its performance. All nanofluids were stored at a temperature of 30 °C and duplicated in an oven at 60 °C. The methods are described in Table 2 [47].

#### 2.2.2. Turbidity Measurements

The turbidity of the nanofluids determines the particle suspension stability. It was measured at 30 °C by the 8237-absorptiometry method using a Hach 2100P turbidimeter (HATCH, Houston, TX, USA). A standard reference suspension (1–10 NTU: accuracy ±0.1%). For higher values, an accuracy of ±10% was used for the turbidimeter calibration.

#### 2.2.3. Viscosity Test

The viscosities of the samples heated at 30 and 60 °C in an oven for 21 days were measured at 30 °C in a DV3TTM rheometer (AMETEK Brookfield, Middleborough, MA, USA) with an Ultra Low Adapter (ULA, µ < 100 cP, Accuracy: ±1.0%, Repeatability: ±0.2%) by changing the spindle depending on the viscosity of the sample (4.24–106 1/s). The uncertainties in the viscosity results were 1% of the reported value, according to the oil standard reference. The nominal viscosities were determined according to the API RP63 standard [48] at 6 rpm, equivalent to 7.3 1/s for the ULA spindle ($\dot{\gamma }$ = 1.224 rpm). This shear rate was selected for a sandstone formation, with shear rate values between 7 and 10 1/s [49]. Finally, Analysis of Variance (ANOVA) was used to evaluate the influence of continuous and categorical variables on the viscosity values of all nanofluids [50].

#### 2.2.4. Rheological Behavior

The rheological behavior of the nanofluids was measured at 30 °C over the range of 1–100 1/s. All the viscosity data exhibit a good fit for the Carreau–Yasuda model [51,52].

(1) $$\mu =\eta_{\infty }+(\eta_{0}-\eta_{\infty }){\left[1+(\lambda \dot{\gamma }{)}^{\alpha }\right]}^{(n-1)/\alpha }$$

This model describes the behavior of non-Newtonian fluids [36] as a function of the zero shear viscosity $\eta_{0}$ (cP), the infinite shear viscosity $\eta_{\infty }$ (cP), the effective shear rate, $\dot{\gamma }$ (1/s), the relaxation parameter λ (s) (which limits the transition zone between dilatant and pseudoplastic behavior), the power law exponent n (dimensionless), and the transition parameter α (dimensionless, describes the transition of the behavior at time zero and the critical point of the shear rate) [53].

## 3. Results

### 3.1. Nanofluid’s Viscosity

**Figure 1:**
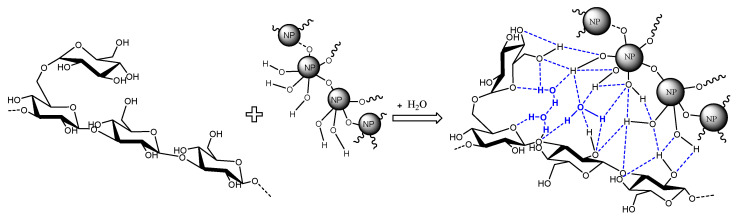
Schematic of the interaction between the SG chain and the hydrophilic NPs in the aqueous phase.

**Figure 2:**
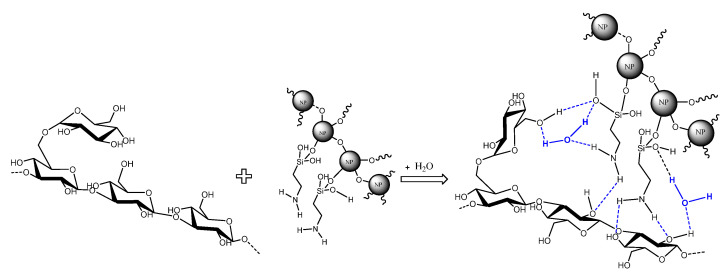
Schematic of the interaction between the SG chain and the SiO_2__APTES_120 NPs in the aqueous phase.

The SG concentration used in all the experiments was set at 1000 ppm, according to the results previously reported by the authors [44,45]. Table 3 displays the viscosity values of the nanofluids prepared by method I. It is observed that the viscosity of the SG solutions and the nanofluids were not affected by temperature (30 and 60 °C, Table 3), which was expected due to the high thermal stability of the biopolymer. Adding all NPs positively affects the viscosity of the SG solutions (increments up to 11.3%, 6.2% average). The increment in viscosity can be attributed to the interactions between the glycosidic groups of the SG with the OH groups on the surface of the NPs through hydrogen bonding (Figure 1) and the hydrophobic interactions between the APTES on the NPs (Figure 2) and the backbone of the biopolymer. Furthermore, the NPs act as crosslinkers between the SG chains.

Despite the viscosity results, the nanofluids containing TiO_2__65, Al_2_O_3__120, Al_2_O_3__180, and Al_2_O_3__35 NPs should not be considered as EOR additives due to their low dispersity into the biopolymer solutions, attributed to the low density of silanol groups on their surface [44,45].

The viscosity values of the nanofluids prepared by method II are presented in Table 4. Adding all NPs to the SG solution increased its viscosity up to 15.2% (8.9% average). At Theating = 30 °C, the NPs with the highest viscosifying effect were alumina Al_2_O_3__120, Al_2_O_3__180, and SiO_2__380. However, Al_2_O_3__120, Al_2_O_3__180, Al_2_O_3__35, and TiO_2__65 NPs were unstable in the SG solution. At Theating = 60 °C, the highest viscosity values were obtained with all SiO_2_ NPs due to their higher stability than the Al_2_O_3_ and TiO_2_ NPs.

Table 5 presents the viscosity values of the nanofluids prepared by method III. Adding the NPs increased the viscosity of the SG solution up to 12.7% (5.4% average, Table 5). As in methods I and II, Al_2_O_3__120, Al_2_O_3__180, Al_2_O_3__35, and TiO_2__65 NPs were unstable in the SG solution.

In method IV, the increments in the SG viscosity by adding the NPs reached up to 10.8% (3.7% average, Table 6). The SG + Al_2_O_3_ and SG + TiO_2_ nanofluids were unstable as in the previous preparation methods.

All of the nanofluids tested have a decrease in viscosity (measured at 30 °C) after heating the samples for 0, 7, 14, and 21 days at 60 °C. This effect is caused by the weakening of the intermolecular forces between the NPs and the SG chains. Furthermore, the agglomeration of the NPs over time increases their particle size, reducing their Brownian velocity. When the Brownian velocity reaches terminal settling velocity, the NPs cannot overcome the gravitational force and precipitate in the nanofluids, causing viscosity reduction over time [54].

By comparing all viscosity data, it can be concluded that the nanofluids prepared by methods I and II exhibited the highest viscosity values. When the NPs are dispersed in the SG solution instead of in water (methods III and IV), the stability of the suspension increases because the viscosity of the dispersion medium is higher (Stoke’s law) [55].

Comparing methods I and II, it is observed that the dispersion of the NPs is affected by the stirring speed. At high stirring speed (method I @ 20,000 rpm × 5 min vs. method II @ 500 rpm × 60 min), the NPs move at the side of the beaker wall without being distributed throughout the biopolymer solution. It reduces the interaction with NP-SG, leading to lower viscosity increments for the nanofluids prepared by method I. Accordingly, method II is the one recommended for preparing SG-based nanofluids.

### 3.2. Nanofluid’s Stability

**Figure A1:**
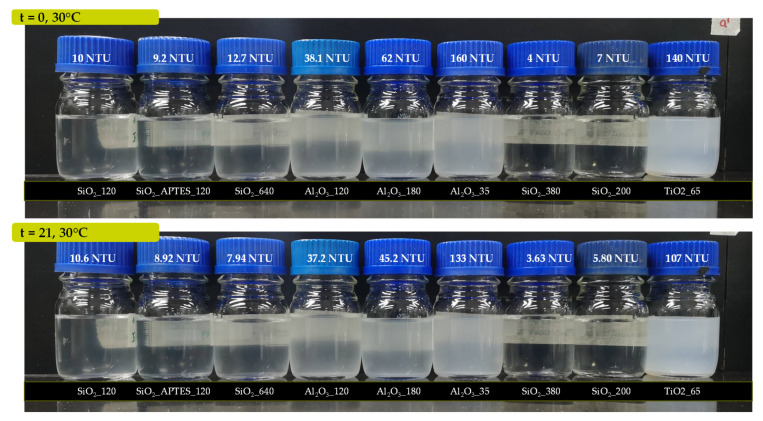
Visual stability test and turbidity values of the nanofluids prepared by method I after heating the samples for 0 and 21 days at 30 °C.

**Figure A2:**
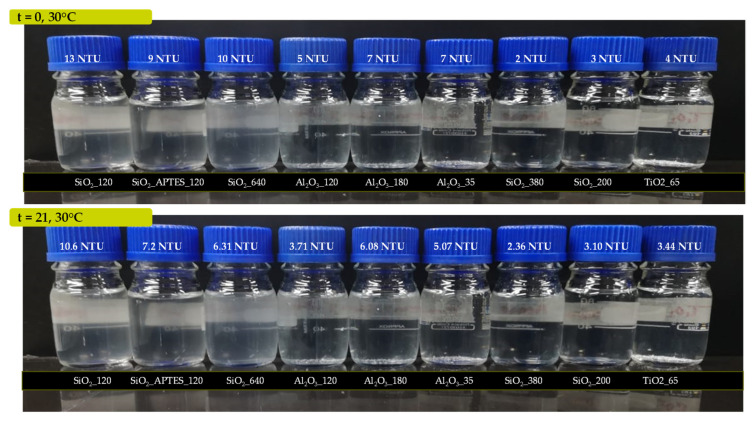
Visual stability test and turbidity values of the nanofluids prepared by method II after heating the samples for 0 and 21 days at 30 °C.

**Figure A3:**
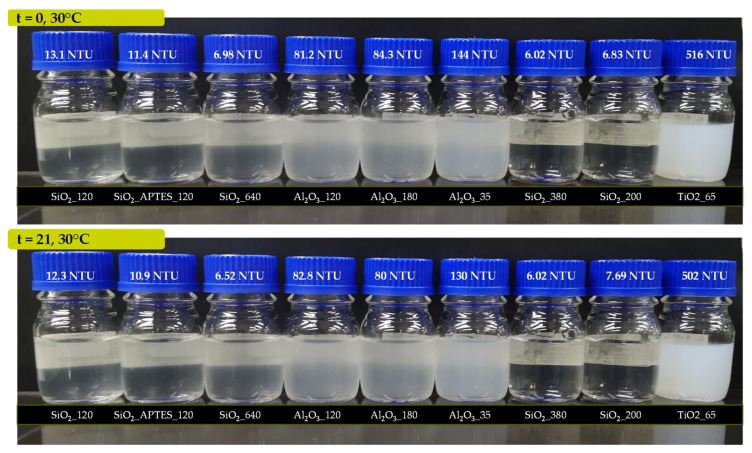
Visual stability test and turbidity values of the nanofluids prepared by method III after heating the samples for 0 and 21 days at 30 °C.

**Figure A4:**
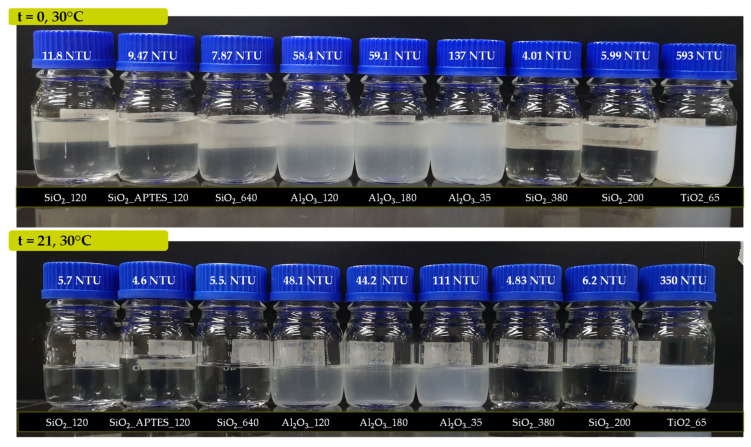
Visual stability test and turbidity values of the nanofluids prepared by method IV after heating the samples for 0 and 21 days at 30 °C.

Visual observation and turbidity measurements were used to study the stability of the SG-based nanofluids. During the visual stability test (see the images of the nanofluids in Appendix A, Figure A1, Figure A2, Figure A3 and Figure A4), the SG + TiO_2_ and SG + Al_2_O_3_ nanofluids exhibited rapid agglomeration and settlement. In contrast, the SG + SiO_2_ nanofluids were stable for more than 21 days.

**Figure A5:**
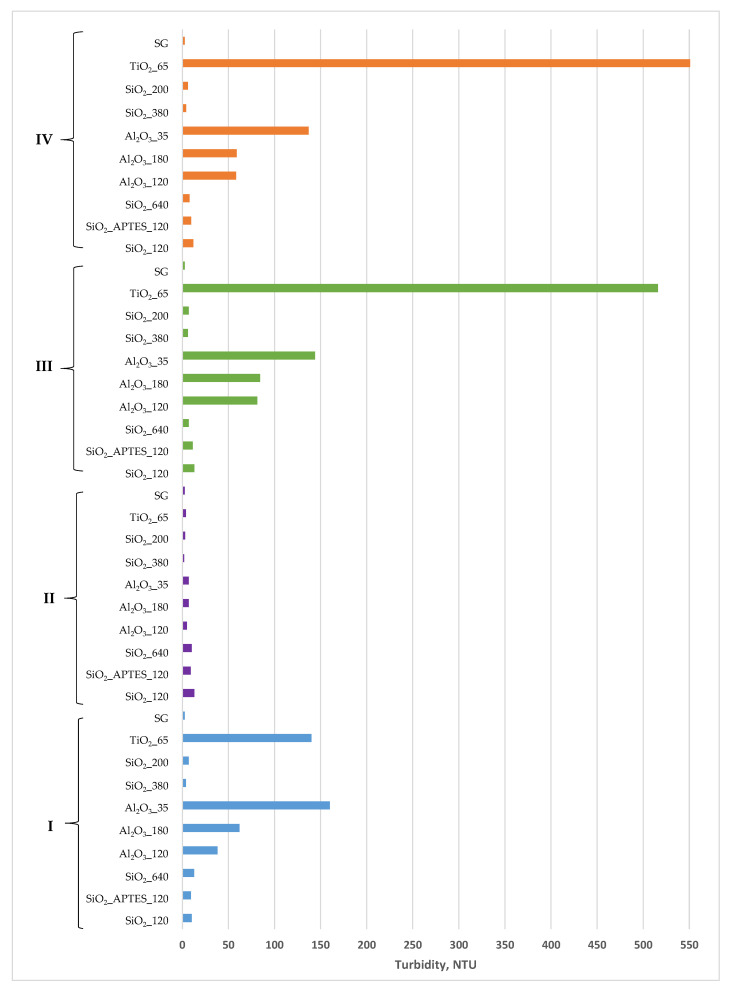
Comparison of the turbidity values of the nanofluids at 0 days and 30 °C.

Accordingly, the SG + SiO_2_ nanofluids exhibited the lowest turbidity values because the good dispersion of the SiO_2_ NPs in the SG solution reduced the amount of light scattered (Table 7). The SG + TiO_2_ and SG + Al_2_O_3_ nanofluids showed the highest turbidity values because the low interaction between the NPs and the SG chains causes the agglomeration of the NPs and higher light scattering in the nanofluid. The SG + TiO_2_ and SG + Al_2_O_3_ nanofluids prepared by method II showed lower turbidity values than the other methods due to the rapid precipitation of the NPs observed during the visual stability test. From these results, it can be concluded that SG + SiO_2_ are the most stable nanofluids, regardless of the preparation method (Appendix A, Figure A5).

### 3.3. Statistical Analysis

**Figure A6:**
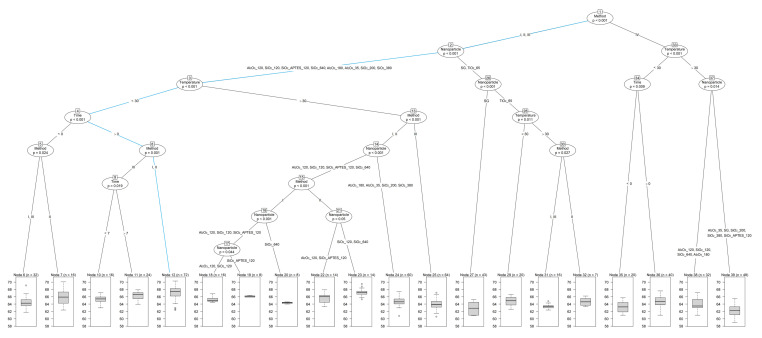
Statistical decision tree.

The statistical decision tree determined the best preparation method and nanofluid (Appendix A, Figure A6). The results show methods I and II, and SG + SiO_2__120 and SG + SiO_2__APTES_120 nanofluids (Figure A6, blue line).

The analysis of the experimental data was conducted following a 2^k^ factorial design using R statistical software (version 4.2.2) to examine the effects. The interactions of preparation method, standing time, heating temperature, and nanoparticle type on the viscosity of the nanofluids within an empirically selected range of high (1) and low (−1) levels are summarized in Table 8. The standing time and heating temperature are continuous variables, while the preparation method and NP type are discrete variables.

**Figure 3:**
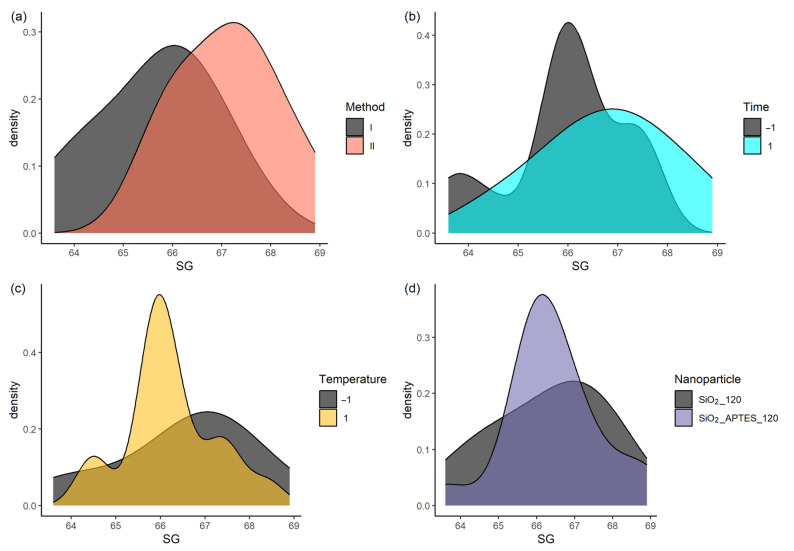
Viscosity as a function of (**a**) preparation method, (**b**) standing time, (**c**) temperature, and (**d**) nanoparticle type.

The highest viscosity values are obtained with method II (Figure 3a) using SiO_2__120 NPs (Figure 3d) after 21 days of standing time (high level, Figure 3b) at 30 °C (low level, Figure 3c).

The previous analysis provided the optimal levels of each factor but could not determine which factors impact the response variable (viscosity) most. To achieve this, an analysis of variance (ANOVA [56]) was performed. According to the ANOVA results (Table 9), the preparation method and standing time influence the nanofluid viscosity with a statistical significance of 95% [50]. On the contrary, the heating temperature and NP type are insignificant (*p* > 0.05) [57].

In this study, it is also observed that nanoparticles SiO_2__120 and SiO_2__APTES_120, spherical with average sizes of 20 nm and a superficial area of 120 m^2^/g, are present in the solutions with higher viscosity. Similarly, nanoparticles SiO_2__640, amorphous porous nanoparticles of 20 nm but with a higher superficial area (640 m^2^/g), generated greater dispersions, increasing the variability in the viscosity data. On the other hand, SiO_2_ spherical nanoparticles of size 20 nm significantly increase viscosity (Figure A6). According to Keblinski et al. [58], a low nanoparticle size (at constant concentration) will augment the viscosity value of nanofluids due to an interparticle spacing decrease, which intensifies interparticle interaction and generates greater aggregate structure.

### 3.4. Rheological Behavior of Nanofluid

**Figure 4:**
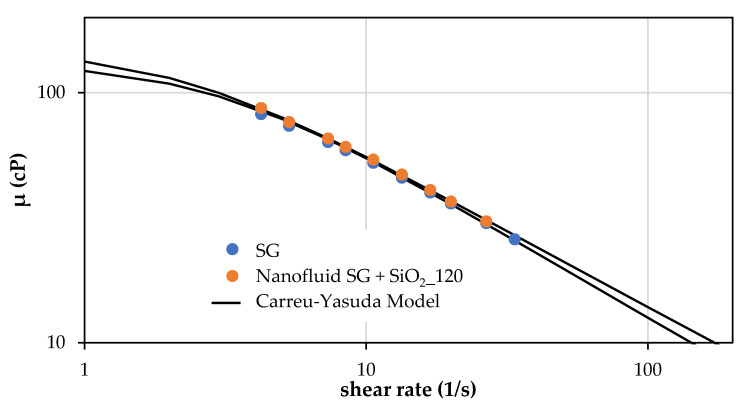
Rheological behavior of the SG and nanofluid SG + SiO_2__120 solutions prepared by method II at 30 °C.

Figure 4 shows that SG and nanofluid SG + SiO_2__120 solutions exhibited a shear thinning behavior with pseudo-plasticity indices (*n*) less than one [59], where hydroxyl groups over silica nanoparticles surface could contribute to improving the interactions with OH groups around the rod-like structure of the Scleroglucan [60].

For a rheological explanation of the increase in bulk viscosity with the addition of the nanofluid prepared by method II, the rheological parameters of the Carreau–Yasuda model, such as relaxation time, viscoelasticity index, and viscosity at zero and infinite time, are essential to establishing the nanoparticle effect. The infinite shear viscosity and the relaxation parameter were fixed at 0.458 cP and 1.5 s, respectively [8]. The Carreau–Yasuda model parameters of SG and nanofluid SG + SiO_2__120 are shown in Table 10. Nanofluid SG + SiO_2__120 viscosity parameters at zero times ($\eta_{0})$ and relaxation times (λ) are higher than the SG solution. The pseudo-plasticity index (*n*) is slightly higher for nanofluid SG + SiO_2__120 due to its higher pseudo-plasticity compared with the SG solution related to the rising in the molecular entanglement [61,62].

## 4. Conclusions

This paper provides insights into the effect of the preparation method and NP type on the stability and viscosity of SG-based nanofluids. The addition of all NPs improved the thickening behavior of the SG solution due to the formation of three-dimensional structures between the NPs and the SG polymeric chains. These structures are formed through hydrogen bonding between the glycosidic groups of the SG and the silanol groups on the surface of the NPs or through hydrophobic interactions between the APTES on the NPs and the backbone of the SG.

From the visual observation and the turbidity monitoring, the SG + SiO_2_ exhibited the lowest turbidity values and good dispersion because hydroxyl groups over the surface of silica nanoparticles could contribute to improving the OH groups interactions with the structure of Scleroglucan, regardless of the preparation method. Furthermore, the SG + Al_2_O_3_ and SG + TiO_2_ nanofluids showed the highest turbidity values because the low interaction between the NPs and the SG chains caused the agglomeration of the NPs and higher light scattering in the nanofluid, attributed to the low density of silanol groups on their NP surface. The selected method for the nanofluid preparation was method II, and the nanofluid with a higher viscosity increment was 1000 ppm of SG + 100 ppm of SiO_2__120 NPs.

Finally, it can be concluded that the nanofluid SG + SiO_2__120 exhibits higher zero-shear viscosity, pseudo-plasticity index, and relaxation times due to its higher molecular entanglement compared with the SG solution.

## Figures and Tables

**Table 1:** Nanoparticles are used to prepare the SG-based nanofluids.

Name	Description	Supplier
SiO_2__120	SiO_2_ (20 nm, 120 m^2^/g, spherical, hydrophilic)	Nanostructured & Amorphous Materials Inc., Los Alamos, NM, USA
SiO_2__APTES_120	SiO_2_ (20 nm, 120 m^2^/g, spherical, amphiphilic, surface coated by (3-aminopropyl) triethoxysilane 2%—APTES	
SiO_2__640	SiO_2_ (20 nm, 640 m^2^/g, amorphous porous)	
Al_2_O_3__120	Al_2_O_3_ (10 nm, 120 m^2^/g, spherical, gamma, hydrophilic)	
Al_2_O_3__180	Al_2_O_3_ (20–30 nm, 180 m^2^/g, nearly spherical, gamma, hydrophilic)	
Al_2_O_3__35	Al_2_O_3_ (27–43 nm, 35 m^2^/g, mainly alpha contains 5–10% gamma, hydrophilic)	
SiO_2__380	SiO_2_ (12–15 nm, 380 m^2^/g, amorphous, hydrophilic)	Evonik industries, Allentown, PA, USA
SiO_2__200	SiO_2_ (12 nm, 200 m^2^/g, amorphous, hydrophilic)	
TiO_2__65	Titanium (IV) oxide (21 nm, 35–65 m^2^/g)	Sigma Aldrich, St. Louis, MO, USA

**Table 2:** Description of the preparation methods of the Scleroglucan-based nanofluids.

Method	Step 1	Step 2	Step 3
I	Dissolve the SG powder into the brine under mechanical stirring at 500 rpm for 10 min. Then, stir the sample at 800 rpm and 40 °C for 10 min. Finally, homogenize the solution for 5 min using a high-performance immersion blender (IKA™ T 25 Digital Ultra-Turrax)	Add the NPs to the SG solution	Stir the nanofluid with the Ultra-Turrax at 20,000 rpm for 5 min
II	Same as described in method I (step 1)	Add the NPs to the SG solution	Stir the nanofluid with the propeller agitator at 500 rpm for 60 min
III	Disperse the NPs in brine and ultrasonicate the dispersions for 1 h	Same as described in method I (step 1)	-
IV	Add the SG powder and the NPs into the brine simultaneously. Stir the sample with a metallic blade for 10 min at 500 rpm. Then, stir the sample at 800 rpm and 40 °C for 10 min. Finally, stir the dispersion with the Ultra-Turrax at 20,000 rpm for 5 min.	-	-

**Table 3:** Viscosity measurements of the SG-based nanofluid prepared by method I at 7.3 s^−1^ and 30 °C after heating the samples for 0, 7, 14, and 21 days at 30 °C and 60 °C.

Heating Temperature (°C)	Sample	Viscosity, cP				Viscosity Changes of the SG Solution			
		0	7	14	21	0	7	14	21
30	SiO_2__120	63.76	67.14	67.94	66.98	4.5%	10.0%	11.3%	9.8%
	SiO_2__APTES_120	63.56	64.66	66.96	67.38	4.2%	6.0%	9.7%	10.4%
	SiO_2__640	63.70	64.44	66.96	66.90	4.4%	5.6%	9.7%	9.6%
	Al_2_O_3__120	63.92	65.62	64.12	67.36	4.8%	7.5%	5.1%	10.4%
	Al_2_O_3__180	61.80	66.90	64.50	67.74	1.3%	9.6%	5.7%	11.0%
	Al_2_O_3__35	64.64	65.20	65.86	66.24	5.9%	6.9%	7.9%	8.6%
	SiO_2__380	62.98	65.70	65.86	67.18	3.2%	7.7%	7.9%	10.1%
	SiO_2__200	65.24	66.22	65.38	67.22	6.9%	8.5%	7.1%	10.2%
	TiO_2__65	63.70	64.70	64.36	65.56	4.4%	6.0%	5.5%	7.4%
	SG	61.02	61.00	62.90	61.70	0.0%	0.0%	3.1%	1.1%
60	SiO_2__120	65.90	64.98	65.02	64.52	8.0%	6.5%	6.6%	5.7%
	SiO_2__APTES_120	65.88	66.38	66.06	65.94	8.0%	8.8%	8.3%	8.1%
	SiO_2__640	64.58	64.40	64.30	64.02	5.8%	5.5%	5.4%	4.9%
	Al_2_O_3__120	64.58	66.80	65.30	64.94	5.8%	9.5%	7.0%	6.4%
	Al_2_O_3__180	64.16	65.06	67.08	65.70	5.1%	6.6%	9.9%	7.7%
	Al_2_O_3__35	63.98	63.98	64.84	64.44	4.9%	4.9%	6.3%	5.6%
	SiO_2__380	64.68	63.80	65.10	63.66	6.0%	4.6%	6.7%	4.3%
	SiO_2__200	64.10	65.44	65.60	64.12	5.0%	7.2%	7.5%	5.1%
	TiO_2__65	62.56	63.32	63.46	63.58	2.5%	3.8%	4.0%	4.2%
	SG	61.02	61.82	62.66	60.88	0.0%	1.3%	2.7%	−0.2%

**Table 4:** Viscosity measurements (cP) of the SG-based nanofluid prepared by method II at 7.3 s^−1^ and 30 °C after heating the samples for 0, 7, 14, and 21 days at 30 °C and 60 °C.

Heating Temperature (°C)	Sample	Viscosity, cP				Viscosity Changes of the SG Solution			
		0	7	14	21	0	7	14	21
30	SiO_2__120	67.5	68.9	69.3	67.6	10.6%	12.9%	13.6%	10.8%
	SiO_2__APTES_120	67.1	68.2	68.6	68.9	10.0%	11.8%	12.4%	12.8%
	SiO_2__640	66.9	67.9	67.5	68.7	9.6%	11.2%	10.7%	12.6%
	Al_2_O_3__120	68.7	68.9	68.0	68.0	12.5%	13.0%	11.4%	11.4%
	Al_2_O_3__180	70.1	70.3	69.7	69.5	14.8%	15.2%	14.2%	13.9%
	Al_2_O_3__35	66.4	68.2	68.0	68.4	8.8%	11.8%	11.5%	12.1%
	SiO_2__380	68.6	69.4	69.7	69.4	12.4%	13.7%	14.3%	13.7%
	SiO_2__200	66.0	67.0	67.1	67.0	8.1%	9.8%	10.0%	9.8%
	TiO_2__65	65.8	65.5	66.6	66.2	7.8%	7.3%	9.2%	8.5%
	SG	61.0	61.0	62.9	61.7	0.0%	0.0%	3.1%	1.1%
60	SiO_2__120	67.5	68.5	68.8	68.4	10.6%	12.3%	12.7%	12.2%
	SiO_2__APTES_120	66.4	65.7	67.5	65.7	8.8%	7.7%	10.6%	7.7%
	SiO_2__640	67.2	66.9	69.5	66.7	10.2%	9.6%	13.9%	9.3%
	Al_2_O_3__120	66.4	65.8	67.9	65.6	8.8%	7.8%	11.3%	7.4%
	Al_2_O_3__180	64.6	63.8	65.8	63.8	5.8%	4.6%	7.8%	4.6%
	Al_2_O_3__35	63.3	63.3	65.2	63.0	3.7%	3.7%	6.9%	3.2%
	SiO_2__380	65.2	65.3	67.3	65.4	6.9%	7.0%	10.3%	7.2%
	SiO_2__200	65.4	65.2	67.4	65.1	7.2%	6.9%	10.5%	6.6%
	TiO_2__65	63.7	64.1	65.9	65.2	4.4%	5.0%	8.0%	6.9%
	SG	61.0	61.2	62.7	60.9	0.0%	0.3%	2.7%	−0.2%

**Table 5:** Viscosity measurements of the SG-based nanofluid prepared by method III at 7.3 s^−1^ and 30 °C after heating the samples for 0, 7, 14, and 21 days at 30 °C and 60 °C.

Heating Temperature (°C)	Sample	Viscosity, cP				Viscosity Changes of the SG Solution			
		0	7	14	21	0	7	14	21
30	SiO_2__120	62.1	66.9	65.7	64.3	1.8%	9.7%	7.7%	5.4%
	SiO_2__APTES_120	63.9	67.9	66.6	67.3	4.7%	11.3%	9.1%	10.3%
	SiO_2__640	64.2	67.4	66.1	66.3	5.2%	10.4%	8.3%	8.7%
	Al_2_O_3__120	63.4	67.4	67.0	67.0	3.9%	10.5%	9.7%	9.7%
	Al_2_O_3__180	63.7	68.3	67.3	66.8	4.4%	12.0%	10.4%	9.5%
	Al_2_O_3__35	64.7	66.7	65.9	66.8	6.0%	9.3%	8.1%	9.5%
	SiO_2__380	64.8	67.8	67.6	68.8	6.2%	11.2%	10.8%	12.7%
	SiO_2__200	63.6	64.6	62.4	63.0	4.3%	5.9%	2.3%	3.3%
	TiO_2__65	62.6	65.4	63.4	65.9	2.6%	7.1%	3.9%	8.0%
	SG	61.0	61.0	62.9	61.7	0.0%	0.0%	3.1%	1.1%
60	SiO_2__120	65.2	63.8	63.2	63.3	6.9%	4.6%	3.6%	3.7%
	SiO_2__APTES_120	64.9	63.9	63.9	64.0	6.3%	4.7%	4.7%	4.9%
	SiO_2__640	64.3	64.3	63.8	62.8	5.4%	5.3%	4.5%	3.0%
	Al_2_O_3__120	63.6	63.9	63.8	62.7	4.2%	4.7%	4.6%	2.7%
	Al_2_O_3__180	63.1	64.3	64.1	62.7	3.3%	5.3%	5.1%	2.7%
	Al_2_O_3__35	63.8	64.8	64.4	63.4	4.6%	6.2%	5.5%	4.0%
	SiO_2__380	63.3	61.5	64.0	62.0	3.7%	0.8%	4.9%	1.6%
	SiO_2__200	63.6	62.2	63.5	62.7	4.3%	1.9%	4.1%	2.8%
	TiO_2__65	62.4	62.9	63.9	63.1	2.2%	3.1%	4.7%	3.5%
	SG	61.0	61.6	62.7	60.9	0.0%	1.0%	2.7%	−0.2%

**Table 6:** Viscosity measurements of the SG-based nanofluid prepared by method IV at 7.3 s^−1^ and 30 °C after heating the samples for 0, 7, 14, and 21 days at 30 °C and 60 °C.

Heating Temperature (°C)	Sample	Viscosity, cP				Viscosity Changes of the SG Solution			
		0	7	14	21	0	7	14	21
30	SiO_2__120	65.7	67.6	65.2	65.9	7.6%	10.8%	6.9%	7.9%
	SiO_2__APTES_120	63.0	64.2	64.0	64.5	3.3%	5.1%	4.9%	5.7%
	SiO_2__640	65.1	64.6	65.3	64.6	6.6%	5.9%	6.9%	5.9%
	Al_2_O_3__120	64.3	65.0	66.5	66.5	5.3%	6.5%	9.0%	9.0%
	Al_2_O_3__180	62.7	64.4	64.7	65.4	2.7%	5.5%	6.0%	7.2%
	Al_2_O_3__35	64.4	64.6	65.9	65.0	5.5%	5.8%	8.1%	6.5%
	SiO_2__380	65.0	64.8	67.6	64.7	6.5%	6.2%	10.8%	6.0%
	SiO_2__200	65.4	66.0	62.4	65.6	7.2%	8.1%	2.3%	7.5%
	TiO_2__65	61.4	63.5	63.4	63.7	0.7%	4.1%	3.9%	4.4%
	SG	61.0	61.0	62.9	61.7	0.0%	0.0%	3.1%	1.1%
60	SiO_2__120	63.5	63.0	62.1	61.4	4.1%	3.3%	1.8%	0.6%
	SiO_2__APTES_120	62.6	60.9	60.4	60.1	2.6%	−0.3%	−1.0%	−1.5%
	SiO_2__640	64.1	62.1	62.0	61.0	5.0%	1.8%	1.5%	0.0%
	Al_2_O_3__120	63.1	62.8	63.9	63.2	3.4%	2.9%	4.8%	3.5%
	Al_2_O_3__180	63.2	63.1	63.5	63.2	3.6%	3.4%	4.0%	3.5%
	Al_2_O_3__35	62.4	61.9	62.0	62.4	2.2%	1.5%	1.6%	2.3%
	SiO_2__380	63.1	61.5	62.2	61.7	3.5%	0.8%	2.0%	1.1%
	SiO_2__200	61.3	62.1	60.3	60.6	0.4%	1.7%	−1.2%	−0.8%
	TiO_2__65	61.1	62.1	61.5	62.9	0.2%	1.7%	0.8%	3.0%
	SG	61.0	60.8	62.7	60.9	0.0%	−0.4%	2.7%	−0.2%

**Table 7:** Turbidity of the SG-based nanofluid a 30 °C.

Method	Sample	Turbidity	
		0 min after Preparation	21 Days after Preparation
I	SiO_2__120	10.0	10.6
	SiO_2__APTES_120	9.2	8.9
	SiO_2__640	12.7	7.9
	Al_2_O_3__120	38.1	37.2
	Al_2_O_3__180	62.0	45.2
	Al_2_O_3__35	160.0	133.0
	SiO_2__380	4.0	3.6
	SiO_2__200	7.0	5.8
	TiO_2__65	140.0	107.0
	SG	2.6	2.7
II	SiO_2__120	13.0	10.6
	SiO_2__APTES_120	9.0	7.2
	SiO_2__640	10.0	6.3
	Al_2_O_3__120	5.0	3.7
	Al_2_O_3__180	7.0	6.1
	Al_2_O_3__35	7.0	5.1
	SiO_2__380	2.0	2.4
	SiO_2__200	3.0	3.1
	TiO_2__65	4.0	3.4
	SG	2.6	2.7
III	SiO_2__120	13.1	12.3
	SiO_2__APTES_120	11.4	10.9
	SiO_2__640	7.0	6.5
	Al_2_O_3__120	81.2	82.8
	Al_2_O_3__180	84.3	80.0
	Al_2_O_3__35	144.0	130.0
	SiO_2__380	6.0	6.0
	SiO_2__200	6.8	7.7
	TiO_2__65	516.0	502.0
	SG	2.6	2.7
IV	SiO_2__120	11.8	5.7
	SiO_2__APTES_120	9.5	4.6
	SiO_2__640	7.9	5.5
	Al_2_O_3__120	58.4	48.1
	Al_2_O_3__180	59.1	44.2
	Al_2_O_3__35	137.0	111.0
	SiO_2__380	4.0	4.8
	SiO_2__200	6.0	6.2
	TiO_2__65	593.0	350.0
	SG	2.6	2.7

**Table 8:** Experiments and response variable.

Experiment	Preparation Method	Standing Time	Heating Temperature	Nanoparticle Type	Viscosity, cP
1	I	−1	−1	SiO_2__120	64.5
2	II	−1	−1	SiO_2__120	65.7
3	I	1	−1	SiO_2__120	66.3
4	II	1	−1	SiO_2__120	67.4
5	I	−1	1	SiO_2__120	65.9
6	II	−1	1	SiO_2__120	67.5
7	I	1	1	SiO_2__120	64.5
8	II	1	1	SiO_2__120	67.1
9	I	−1	−1	SiO_2__120	63.8
10	II	−1	−1	SiO_2__APTES_120	67.5
11	I	1	−1	SiO_2__APTES_120	67
12	II	1	−1	SiO_2__APTES_120	67.6
13	I	−1	1	SiO_2__APTES_120	65.9
14	II	−1	1	SiO_2__APTES_120	67.5
15	I	1	1	SiO_2__APTES_120	64.5
16	II	1	1	SiO_2__APTES_120	68.4
17	I	−1	−1	SiO_2__120	66.3
18	II	−1	−1	SiO_2__120	65.8
19	I	1	−1	SiO_2__120	67.06
20	II	1	−1	SiO_2__120	68.5
21	I	−1	1	SiO_2__120	65.9
22	II	−1	1	SiO_2__120	66.4
23	I	1	1	SiO_2__120	65.9
24	II	1	1	SiO_2__120	66.2
25	I	−1	−1	SiO_2__120	63.6
26	II	−1	−1	SiO_2__APTES_120	67.1
27	I	1	−1	SiO_2__APTES_120	67.4
28	II	1	−1	SiO_2__APTES_120	68.9
29	I	−1	1	SiO_2__APTES_120	65.9
30	II	−1	1	SiO_2__APTES_120	66.4
31	I	1	1	SiO_2__APTES_120	65.9
32	II	1	1	SiO_2__APTES_120	65.7

**Table 9:** ANOVA results.

Item	Degree of Freedom	Sum of Squares	Mean Square	F-Value	*p*-Value
Preparation method	1	17.024	17.024	15.882	0.00046
Standing time	1	5.009	5.009	4.673	0.03967
Temperature	1	0.738	0.738	0.689	0.41391
Nanoparticle type	1	0.108	0.108	0.101	0.75324
Residual	27	28.94	1.072		

**Table 10:** Carreau–Yasuda model parameters of SG and nanofluid SG + SiO_2__120 at 30 °C.

Parameter	SG Solution	Nanofluid SG + SiO_2__120
$\eta_{0}$ (cP)	132.36	149.22
λ (s)	0.3358	0.4607
n	0.3530	0.3728

## Data Availability

The data presented in this study are available on request from the corresponding author.
